# Transcriptional profiling of human smooth muscle cells infected with gingipain and fimbriae mutants of *Porphyromonas gingivalis*

**DOI:** 10.1038/srep21911

**Published:** 2016-02-24

**Authors:** Boxi Zhang, Allan Sirsjö, Hazem Khalaf, Torbjörn Bengtsson

**Affiliations:** 1 Department of Clinical Medicine, School of Health Sciences, Örebro University, Örebro, Sweden

## Abstract

*Porphyromonas gingivalis* (*P. gingivalis*) is considered to be involved in the development of atherosclerosis. However, the role of different virulence factors produced by *P. gingivalis* in this process is still uncertain. The aim of this study was to investigate the transcriptional profiling of human aortic smooth muscle cells (AoSMCs) infected with wild type, gingipain mutants or fimbriae mutants of *P. gingivalis*. AoSMCs were exposed to wild type (W50 and 381), gingipain mutants (E8 and K1A), or fimbriae mutants (DPG-3 and KRX-178) of *P. gingivalis.* We observed that wild type *P. gingivalis* changes the expression of a considerable larger number of genes in AoSMCs compare to gingipain and fimbriae mutants, respectively. The results from pathway analysis revealed that the common differentially expressed genes for AoSMCs infected by 3 different wild type *P. gingivalis* strains were enriched in pathways of cancer, cytokine-cytokine receptor interaction, regulation of the actin cytoskeleton, focal adhesion, and MAPK signaling pathway. Disease ontology analysis showed that various strains of *P. gingivalis* were associated with different disease profilings. Our results suggest that gingipains and fimbriae, especially arginine-specific gingipain, produced by *P. gingivalis* play important roles in the association between periodontitis and other inflammatory diseases, including atherosclerosis.

Atherosclerosis is a pathologic process, which associates with atheromatous plaque formation in the inner lining of the arteries. This process is a slow phenomenon, starts in early age and progresses during life until revealing of the clinical symptoms^1^. From the beginning, atherosclerosis was considered as an abnormal accumulation of lipids in the artery wall, however, it is well established nowadays that different immunological and inflammatory processes within the artery wall play a key role in the pathogenesis of atherosclerosis^2^.

During the development of atherosclerosis, a variety of cell types are involved in this complicated process, such as endothelial cells, smooth muscle cells (SMCs), T cells, macrophages, and monocytes. Arterial endothelial cells, which compose the inner arterial surface, resist white blood cells attachment under healthy condition. However, during the atherosclerotic process, endothelial cells that are stimulated by cytokines and oxidized lipids become permeable, leading to the infiltration of monocytes and T lymphocytes into the vascular intima^3^. In the intima, the monocytes internalize oxidized low-density lipoprotein (LDL) to form foam cells, which further results in the production of cytokines that activate SMCs. The activated SMCs then migrate from the media into the intima followed by elevated proliferation^4^. SMCs are the main source of extracellular matrix molecules, which form the fibrous cap that covers the plaque and contributes to the development of atherosclerosis^5^.

High circulating levels of LDL, smoking, and low physical activity have been considered as risk factors that are associated with the development of atherosclerosis^6^. Besides those factors, periodontal disease was identified as a novel risk factor of cardiovascular disease, and in particular, atherosclerosis^7^,^8^. The DNA of *P. gingivalis* have been found in human atherosclerotic lesions^9^. Although, it is hard to conduct experiments on human directly, animal experiments have shown that *P. gingivalis* infection can trigger and accelerate the formation of coronary and aortic atherosclerosis^10^,^11^.

The severity of periodontitis significantly correlates to the concentrations of *P. gingivalis* in periodontal pockets^12^. The virulence factors, such as lipopolysaccharides (LPS), fimbriae, capsule, haemagglutinins and proteases (gingipains), harbored by *P. gingivalis* enhance the biofilm formation and are linked to the development of atherosclerosis^13^. Gingipains are cysteine proteases, which include arginine gingipains (Rgp) and lysine gingipain (Kgp). Gingipains, the main virulence factors produced by *P. gingivalis*, modulate the expression of cytokines and immunoglobulins and thus affect the immune responses of the host cells^14^,^15^,^16^,^17^. Fimbriae are hair-like protein structures of the outer surfaces of bacteria. Studies have shown that fimbriae facilitate bacteria to attach and invade to the host cells^18^,^19^. There are two groups of fimbriae produced by *P. gingivalis*, major fimbriae and minor fimbriae, and both are involved in the development of periodontitis^20^,^21^.

Up to now, no study has been conducted to elucidate the signaling mechanisms of the virulence factors during *P. gingivalis* infection of SMCs, and thereby their role in atherosclerosis. The aim of this study was to investigate the effects of gingipains and fimbriae in the regulation of gene expression profiling in human aortic smooth muscle cells.

## Results

### Distinct gene lists regulated by different strains of *P. gingivalis*

The microarray experiments were performed to analyze the gene expression in AoSMCs treated with different *P. gingivalis* strains, including ATCC33277 (wild type), W50 (wild type), 381 (wild type), E8 (W50 derived Rgp mutant), K1A (W50 derived Kgp mutant), DPG3 (381 derived major fimbriae mutant), and KRX178 (381 derived minor fimbriae mutant). Through analyzing microarray raw data using limma package, we got 7 lists of differentially expressed genes of interest based on setting the threshold of fold change >1.5 with adjust *p-value* (Benjamini-Hochberg) <0.05. The wild type and gingipain mutants infected groups were compared to uninfected control group and the fimbriae mutants infected groups were compared to erythromycin treated group. The wild type *P. gingivalis* strains, ATCC33277 (Table S1), W50 (Table S2), and 381 (Table S3) showed more power to regulate the gene expression than E8 (Table S4), K1A (Table S5), DPG3 (Table S6), and KRX178 (Table S7) in AoSMCs.

### Disease ontology (DO) analysis for genes differentially expressed from AoSMCs infected with *P. gingivalis*

**Figure 1:**
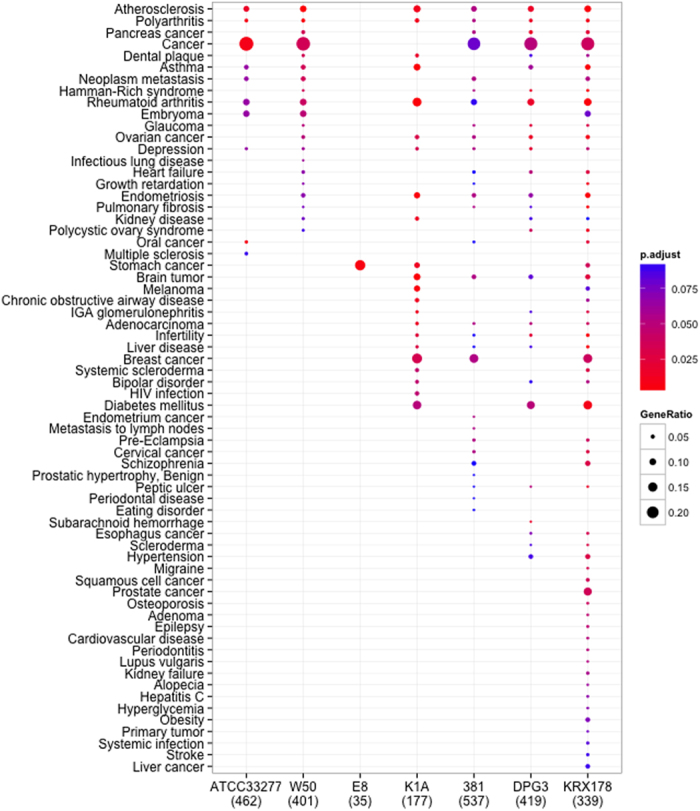
Disease ontology analysis for genes differentially expressed by AoSMCs infected with *P. gingivalis*. The enriched diseases by differentially expressed genes regulated by wild type (ATCC33277, W50, and 381), Rgp mutant (E8), Kgp mutant (K1A), major fimbriae mutant (DPG3), and minor fimbriae mutant (KRX178) *P. gingivalis* in AoSMCs were analyzed using clusterProfiler R package with adjust *p-value* < 0.1.

To identify the differentially expressed genes correlated diseases, DO analysis was carried out by input the Entrez Gene identifiers (Entrez Gene IDs) from the gene lists into *clusterProfiler* package. We found that the differentially expressed genes regulated by *P. gingivalis* strain ATCC33277 were enriched in less DO categories compared to other wild type strains W50 and 381. In addition, for AoSMCs infected with *P. gingivalis* strain KRX178, the largest number of DO categories were found compared to other strains. However, the Rgp mutants E8 only significantly correlated to one DO term. (Fig. 1) The summary of DO analysis was list on Table S8.

### Functional analysis for genes differentially expressed from AoSMCs infected with *P. gingivalis* gingipain mutants

**Figure 2:**
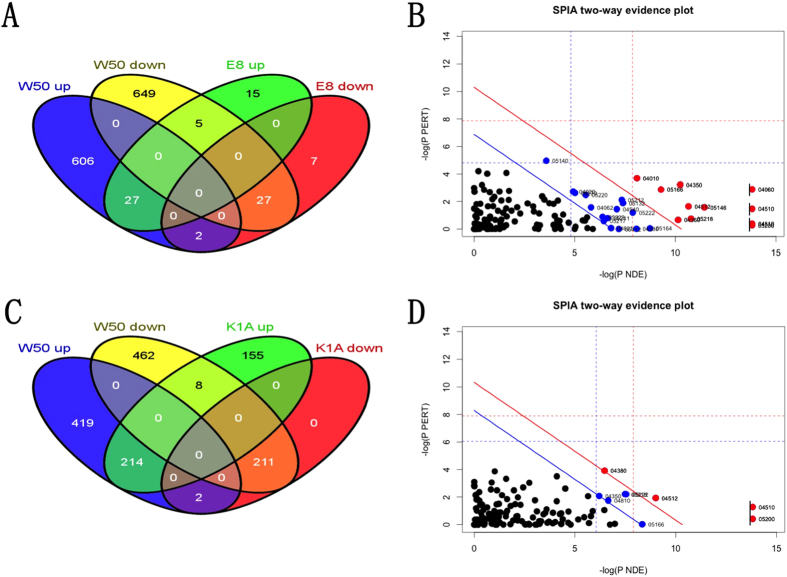
(A–D) Functional analysis for genes differentially expressed by AoSMCs infected with *P. gingivalis* gingipain mutants. Venn diagrams showing the number of wild type *P. gingivalis* W50 up-regulated (W50 up) and down-regulated (W50 down) genes compared with Rgp mutant *P. gingivalis* up-regulated (E8 up) and down-regulated (E8 down) genes (**A**) and Kgp mutant *P. gingivalis* up-regulated (K1A up) and down-regulated (K1A down) genes (**C**) in AoSMCs. SPIA analysis results depicting the enriched KEGG pathways for uncommon genes between W50 and E8 (**B**), or uncommon genes between W50 and K1A (**D**). Each dot refers to a KEGG pathway with the KEGG pathway ID. The blue line indicates the significant level of 5% with Benjamini-Hochberg FDR correction. The red line indicates the significant level of 5% with Bonferroni correction.

From the wild type W50 and W50-derived Rgp gingipain mutant E8 infected AoSMCs, we found 54 common differentially express genes (Fig. 2A). In order to understand the roll of Rgp in the process of *P. gingivalis* infection of AoSMCs, the uncommon expression genes were picked out and insert into R platform and analyzed by SPIA package. These uncommon genes were significantly enriched in 27 KEGG pathways, including focal adhesion pathway, NOD-like receptor signaling pathway, MAPK signaling pathway, TGF-beta signaling pathway and several pathways related to different cancers (Fig. 2B, Table S9). The SPIA analysis of the uncommon differentially expressed genes (Fig. 2C) between the wild type W50 and W50-derived Kgp gingipain mutant K1A infected AoSMCs indicated that 9 KEGG pathways were significantly enriched, which are all included in KEGG pathways that derived from the uncommon genes when comparing W50 regulated genes to E8 regulated genes in AoSMCs (Fig. 2D, Table S9).

### Functional analysis for genes differentially expressed from AoSMCs infected with *P. gingivalis* fimbriae mutants

**Figure 3:**
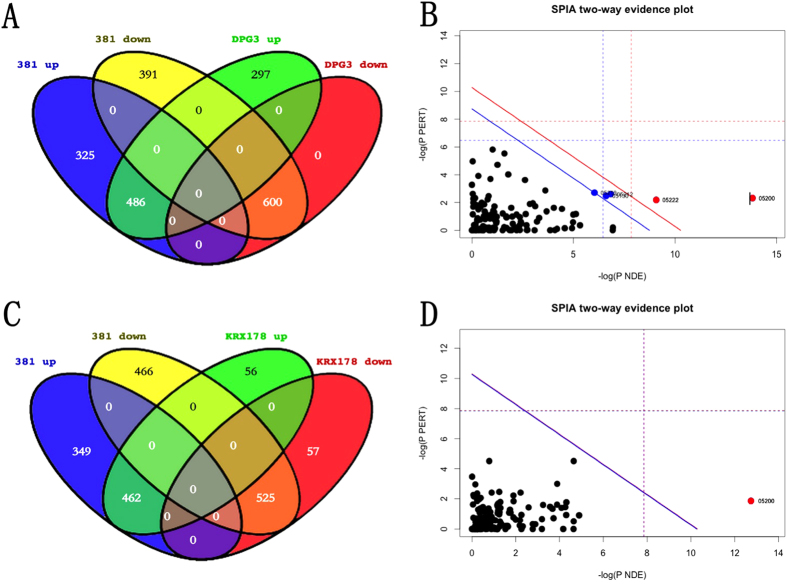
(A–D) Functional analysis for genes differentially expressed by AoSMCs infected with *P. gingivalis* fimbriae mutants. Venn diagrams showing the number of wild type *P. gingivalis* W50 up-regulated (W50 up) and down-regulated (W50 down) genes compared with major fimbriae mutant *P. gingivalis* up-regulated (DPG3 up) and down-regulated (DPG3 down) genes (**A**) and minor fimbriae mutant *P. gingivalis* up-regulated (KRX178 up) and down-regulated (KRX178 down) genes (**C**) in AoSMCs. SPIA analysis results depicting the enriched KEGG pathways for uncommon genes between W50 and DPG3 (**B**), and uncommon genes between W50 and KRX178 (**D**). Each dot refers to a KEGG pathway with the KEGG pathway ID. The blue line refers to significant level of 5% with Benjamini-Hochberg FDR correction. The red line refers to significant level of 5% with Bonferroni correction.

Comparing the wild type *P. gingivalis* strain 381 with its corresponding major fimbriae mutant DPG3, the differentially expressed uncommon genes (Fig. 3A) were significantly enriched in 5 pathways, which include pathways in cancer, small cell lung cancer, pancreatic cancer, pathogenic Escherichia coli infection, and bladder cancer (Fig. 3B, Table S10). There are less differentially expressed genes between 381 and KRX178 (Fig. 3C) than the differentially expressed genes between 381 and DPG3. Only one KEGG pathway, related to cancer, was significantly enriched by those uncommon genes (Fig. 3D, Table S10).

### Functional analysis for genes differentially expressed from AoSMCs infected with wild type *P. gingivalis*

**Figure 4:**
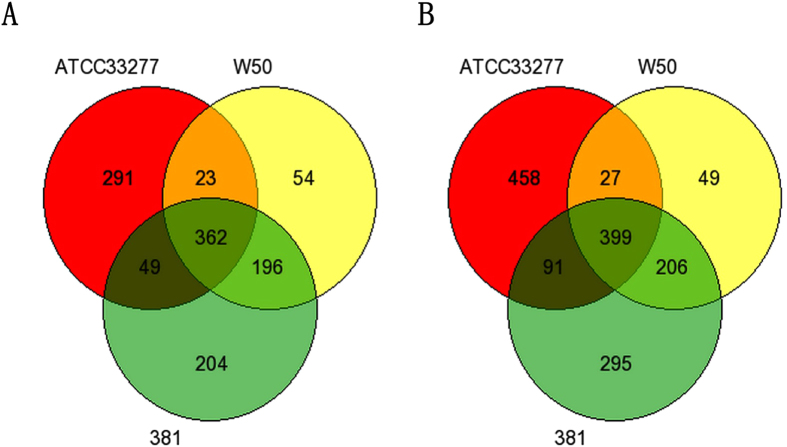
(A,B) Venn diagrams for differentially expressed genes in AoSMCs stimulated with different wild type *P. gingivalis*. Venn diagrams revealing the number of genes significantly up-regulated (**A**) or down-regulated (**B**) by wild type *P. gingivalis* strains ATCC33277, W50, and 381.

The venn diagrams for the differentially expressed genes from each wild type *P. gingivalis* strain treated groups were showed in Fig. 4. To find the key functions that are targeted by *P. gingivalis* infection, we did the gene ontology analysis for the common genes with significantly up-regulated and down-regulated genes, respectively, using Integrated Discovery (DAVID) bioinformatics tool. The top gene ontology (GO) annotation cluster associate to common up-regulated genes was related to blood vessel development, vasculature development, blood vessel morphogenesis, and the angiogenesiss (Table 1). The top GO annotation cluster coordinates to common down-regulated genes were related to enzyme linked receptor protein signaling pathway, transmembrane receptor protein tyrosine kinase signaling pathway, and cell surface receptor linked signal transduction (Table 1). These results indicated that *P. gingivalis* not only modulates the growth and angiogenesis of AoSMCs, but also alter the cell surface receptors of the cells.

**Figure 5:**
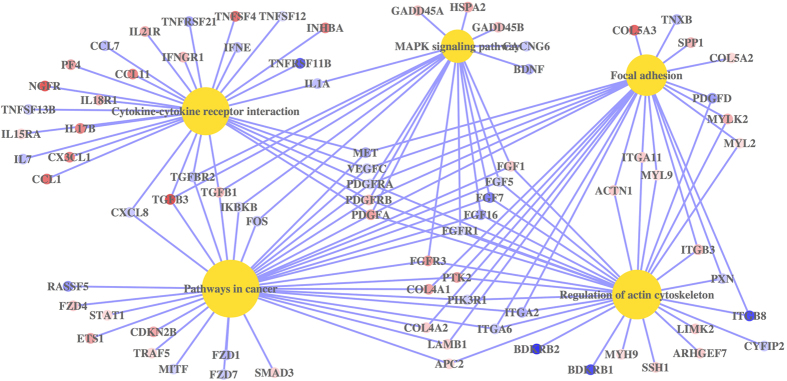
Pathway analysis for common differentially expressed genes regulated by wild type *P. gingivalis* strains. Concept-and-Gene network of enriched KEGG pathways for common differentially expressed genes regulated by wild type *P. gingivalis* strains, was constructed by Bioconductor package GeneAnswers. The KEGG pathways were showed as yellow dot; the up-regulated genes were showed as red dot; the down-regulated genes were showed as blue dot.

To examine the pathways that were enriched by the common regulated genes, the corresponding mean fold change of those genes were inserted into the R platform and analyzed using GeneAnswers package. These genes were enriched in 5 KEGG pathways, which include: pathway in cancer, regulation of actin cytoskeleton, cytokine-cytokine receptor interaction, focal adhesion, and MAPK signaling pathway (Fig. 5).

### Analysis of genes associated with atherosclerosis

**Figure 6:**
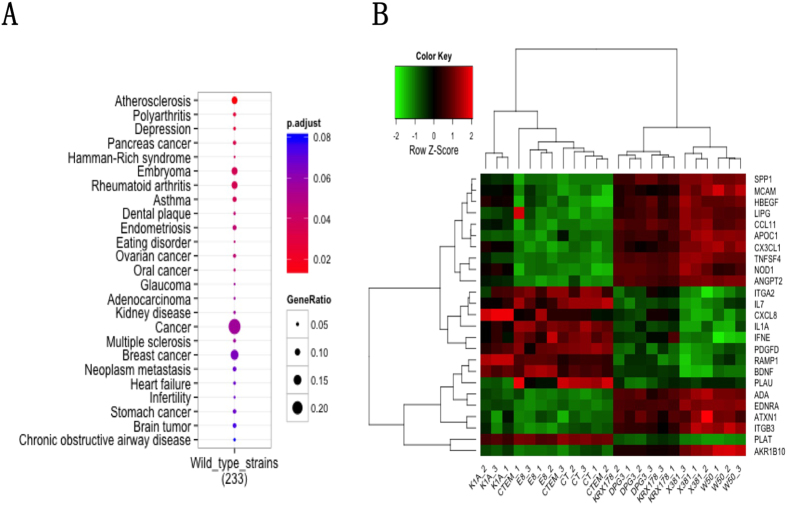
(A,B) Disease ontology for common differentially expressed genes regulated by three wild type *P. gingivalis* strains and genes associated with atherosclerosis. Disease ontology analysis was utilized for common differentially expressed genes regulated by three wild type *P. gingivalis* strains and genes using Bioconductor package clusterProfiler (**A**). Heat map showing expression of genes associated with atherosclerosis in disease ontology (**B**).

The DO analysis for the common differentially expressed genes regulated by the three wild types *P. gingivalis* strains in AoSMCs showed that 27 diseases were significantly enriched and atherosclerosis is identified as the first disease with highest gene ratio (Fig. 6A). 25 genes from the common differentially expressed genes were associated with atherosclerosis (Table S11). The heat map based on the gene expression level from microarray results of those 25 genes revealed that E8 was clustered together with negative control samples and control samples with erythromycin. The fimbriae mutants DPG3 and KRX178 were clustered closer to wild type *P. gingivalis* compared with gingipain mutant K1A that was clustered close to E8 and control samples (Fig. 6B).

### qRT-PCR validation

**Figure 7:**
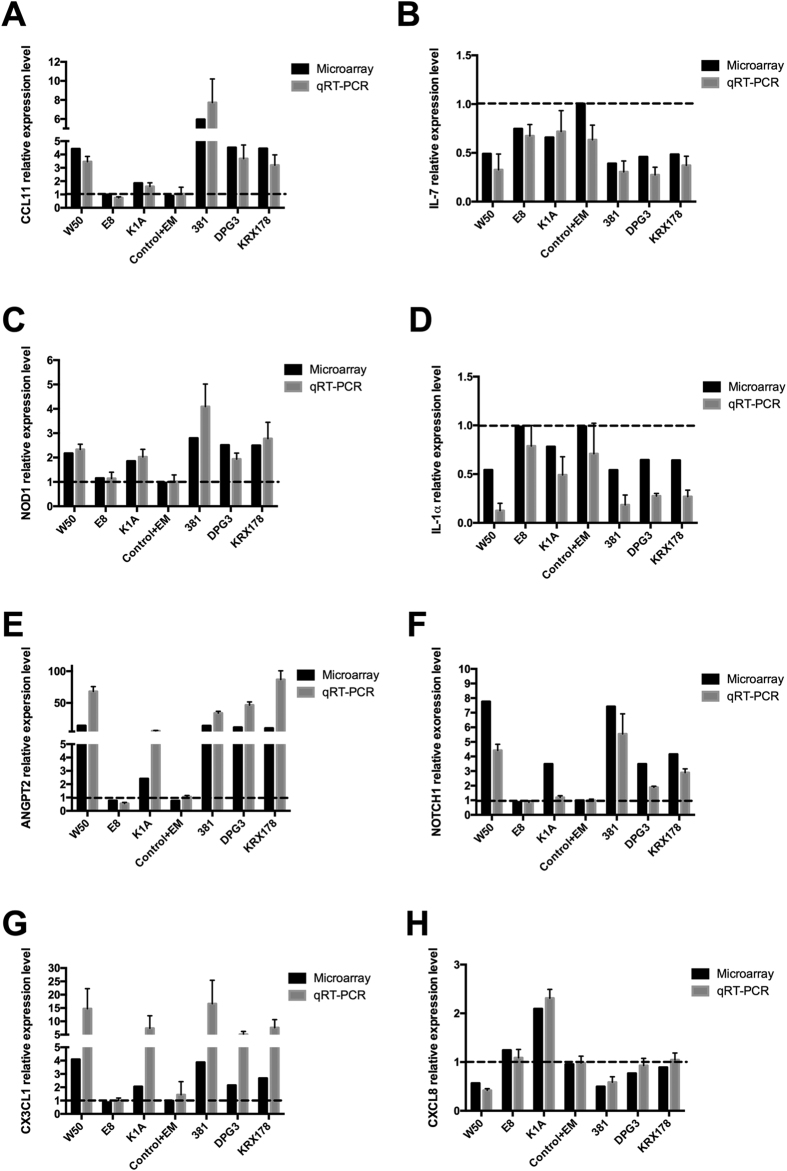
qRT-PCR results for genes of interest in AoSMCs stimulated with *P. gingivalis*. Quantitative real-time PCR results demonstrate relative transcription fold change for CCL11 (**A**), IL-7 (**B**), NOD1 (**C**), IL-1α (**D**), ANGPT2 (**E**), NOTCH1 (**F**), CXCL1 (**G**), and CXCL8 (**H**) of AoSMCs stimulated with wild type (W50 and 381), Rgp mutant (E8), Kgp mutant (K1A), major fimbriae mutant (DPG3), minor fimbriae mutant (KRX178), medium containing 1 ug/ml of erythromycin (Control + EM) as control for fimbriae mutants, or unstimulated with *P. gingivalis* (Control) for 24 h. All these results were normalized with the gene expression level of GAPDH. Fold change was calculated based on negative control for wild type *P. gingivalis* strains and gingipain mutants. For fimbriae mutants, fold change was calculated based on group Control + EM. n = 3.

To validate the results from microarray experiment, we performed qRT-PCR for 8 genes of interest. Among those 8 genes, 7 genes, which include C-C motif chemokine 11 (CCL11), Interleukin 7 (IL-7), Nucleotide-binding oligomerization domain-containing protein 1 (NOD1), Interleukin-1 alpha (IL-1α), Angiopoietin 2 (Angpt2), Fractalkine (CX3CL1), and Interleukin-8 (CXCL8) were showed correlation to the development of atherosclerosis from the DO analysis. We also checked the gene expression of Notch homolog 1 (NOTCH1), which has been found to play an important role in pathogenesis of atherosclerosis^22^,^23^. All qRT-PCR results were proved to have similar regulation as the microarray data (Fig. 7).

## Discussion

Atherosclerosis begins with damage to the endothelium and different traditional risk factors are considered to affect this pathogenic process, such as hypercholesterolemia, smoking and hypertension. In addition, different virus and bacteria have been suggested to be involved in the progress of atherosclerosis^24^. These microorganisms either infect the vascular cells directly or affect the vascular wall indirectly by stimulating other types of cells to produce cytokines and acute phase proteins. Buhlin K, *et al.*^25^ concluded that several traditional risk factors of atherosclerosis correlate to severe periodontitis. M. Yakob, *et al.*^26^ found that *P. gingivalis* infection contributes to the development of carotid atherosclerosis. In this study, we have investigated the role of different virulent factors, which include arginine and lysine gingipains and major and minor fimbriae in *P. gingivalis*-induced inflammation in AoSMCs using microarray technique. Gingipains, which accounts for 85% of the total proteolytic activity of *P. gingivalis*, play multiple roles in regulation of bacterial biofilm formation and host immune responses^27^,^28^. In addition to gingipains, the fimbriae facilitate the binding of *P. gingivalis* to host cells and induce the production of various cytokines^29^,^30^.

In this study, we found that both gingipains and fimbriae affected the differently expressed genes in AoSMCs induced by *P. gingivalis* infection. The gingipain mutants, E8 and K1A, compared with its corresponding wild type strain W50, and the fimbriae mutants, DPG3 and KRX178, compared with its corresponding wild type strain 381, showed deceased number of differently expressed genes. These results reveal that gingipains play an important role in *P. gingivalis* infection of AoSMCs. By utilizing DO analysis, we further explored that how the differentially expressed genes correlated to different diseases. For all *P. gingivalis* strains, except E8, atherosclerosis is highly enriched. In addition, we also found that rheumatoid arthritis (RA) is enriched, which is consistent with previous studies showing that periodontitis and *P. gingivalis* infection are associated with RA^31^,^32^. The DO analysis of the common differentially expressed genes in AoSMCs trigged by wild type *P. gingivalis* also showed that atherosclerosis and RA are significantly enriched. These findings further support an association between pathogens of periodontitis and increased risk of getting other inflammatory diseases.

The SPIA analysis for uncommon genes regulated by gingipain mutants and their corresponding wild type *P. gingivalis* strain showed that both Rgp and Kgp affect the *P. gingivalis*–mediated activation of focal adhesion, ECM-receptor interaction, and actin cytoskeleton pathway. These results are consistent with the pathway analysis for the common differentially expressed genes regulated by wild type *P. gingivalis*, showing that the focal adhesion and the regulation of actin cytoskeleton pathway are enriched. Studies have demonstrated that changes in the interaction between cell and extra cellular matrix (ECM) lead to modulation of the cytoskeleton, which further affect the motility of cells^33^. Remodeling of actin filaments, focal contacts and ECM contributes to the switch of smooth muscle cell phenotype and angiogenesis^34^,^35^. These findings are further consistent with the GO analysis results revealing that the common up-regulated genes regulated by wild type *P. gingivalis* strains in AoSMCs are linked to blood vessel development and angiogenesis. Furthermore, the remodeling affects both proliferation and migration of the smooth muscle cells, which are involved in the process of atherosclerosis^36^,^37^. In a recent study, injection of *P. gingivalis* in rabbit model induces atherosclerosis with the activation of MAPK pathway and the production of cytokines^38^. Accordingly, our pathway analysis for the common differentially expressed genes regulated by wild type *P. gingivalis* reveals that the MAPK pathway and the cytokine-cytokine receptor interaction pathway are enriched. The activation of MAPK cascades is important for vascular smooth muscle cells in neointima formation after vascular injury^39^. In our previous study, we reported that *P. gingivalis* inhibits the inflammatory response in T cells through activation of the MAPK signaling pathway^40^. In this study, we found CXCL8 is down regulated by wild type *P. gingivalis*.

Besides atherosclerosis and rheumatoid arthritis, periodontal disease is also linked to orodigestive cancers^41^. Oral squamous cell carcinoma (OSCC) is one of the most common cancers worldwide and has showed a direct association with *P. gingivalis* infection^42^,^43^. Elevated level of this pathogen was found in gingival carcinomas, compared with healthy gingival tissue. In a large European Prospective Investigation in a Cancer cohort, *P. gingivalis* was associated with more than 2 fold higher risk of pancreatic cancer^44^. In a NHANES cohort study, *P. gingivalis* was linked to a >2 fold increase in risk of orodigestive cancer mortality even independently of clinically appearance of periodontal disease^45^. In this study, we found the among common differentially expressed genes regulated by wild type *P. gingivalis* several are enriched in different cancers, including pancreas cancer and oral cancer. In addition, the function analysis for gingipain and fimbriae mutants revealed that gingipains and fimbriae play a role in the association between *P. gingivalis* infection and orodigestive carcinogenesis. For some cancers, such as OSCC, there is still lack of reliable diagnostic biomarkers and tools, so that the therapies fail to prevent malignant progression^46^. The differentially expressed genes related to cancers in *P. gingivalis*-infected AoSMCs, reported in this study, may suggest possible biomarkers to be used in identification and diagnosis of specific cancers.

Of specific interest was to analyze *P. gingivalis* modulated genes involved in atherosclerosis. Based on DO analysis, we found that 25 genes were identified to correlate with atherosclerosis. The heat map based on these 25 genes, showed no clear difference among negative control groups and the E8-treated group. K1A-stimualted AoSMCs was clustered close to these three groups and the fimbriae mutants DPG3- and KRX178-treated group were clustered close to the wild type *P. gingivalis*, which further proved that gingipains, especially, Rgp are important for *P. gingivalis*-induced inflammation in AoSMCs. We picked out 7 genes from these 25 genes and validated by qRT-PCR. The qRT-PCR results were in good agreement with microarray data and results from unpublished studies. In our previous study, we found that wild type *P. gingivalis* ATCC33277, W50, and 381 up-regulate angiopoietin 2 (Angpt2) in AoSMCs through its corresponding transcription factor, v-ets avian erythroblastosis virus E26 oncogene homolog 1 (ETS1)^47^. The specific function of the genes related to atherosclerosis needs further investigation.

## Conclusion

In summary, this study suggests that *P. gingivalis* infection in AoSMCs is related to many diseases, including atherosclerosis, rheumatoid arthritis, and different forms of cancers. Our findings further reveal possible mechanisms involved in the association between periodontitis and atherosclerosis. Gingipains and fimbriae, especially arginine-specific gingipain Rgp produced by *P. gingivalis,* play a crucial role in *P. gingivalis* infection of AoSMCs. Thus, inhibition of Rgp may be a preventive and therapeutic approach against periodontitis and its associated systemic diseases.

## Methods

### Culture of SMCs

Human primary AoSMCs (Invitrogen, Stockholm, Sweden) were growing in 75 cm^2^ explants culture flasks (Aveen Warner, Limhamn, Sweden) until 80% confluent in n cell culture incubator at 37 °C with 5% CO2 and 95% air using 231 smooth muscle cell culture medium (Gibco, Carlsbad, CA) containing recommended cell growth supplements.

### *P. gingivalis* culture and preparation

W50 (wild type) and its isogenic mutant strains: E8 (Rgp mutant strain); K1A (Kgp mutant), kind gifts from Dr. M. Curtis (Barts and The London, Queen Mary’s School of Medicine and Dentistry, UK), and *P. gingivalis* 381 (wild type), with its corresponding fimbriae mutant strains: DPG3 (major fimbriae mutant) and KRX178 (minor fimbriae mutant), kind gifts from Prof. Genco RJ and Prof. Sharma A. (School of Dental Medicine, University at Buffalo, State University of New York, United States of America), were grown in fastidious anaerobe broth (29.7 g/liter, pH 7.2). For DPG3 and KRX178, the culture medium supplemented with 1 ug/ml erythromycin in plus. All *P. gingivalis* strains were cultured using the anaerobic chamber (80% N_2_, 10% CO_2_, and 10% H_2_, 37 °C) (Concept 400 Anaerobic Workstation; Ruskinn Technology Ltd., Leeds, United Kingdom).

All *P. gingivalis* strains were allowed to growth for 72 h before harvested by centrifugation for 10 min at 10000 rpm at room temperature, washed with Krebs-Ringer-Glucose (KRG) buffer (120 mM NaCl, 4.9 mM KCl, 1.2 mM MgSO_4_, 1.7 mM KH_2_PO_4_, 8.3 mM Na_2_HPO_4_, 10 mM glucose and 1.1 mM CaCl_2_, PH 7.3) and re-suspended in fresh KRG buffer.

The concentration of *P. gingivalis* was determined by counting CFU (Colony-forming unit) of different dilutions of bacteria on fastidious anaerobic agar plate (Acumedia, Neogen, Lansing, USA) enriched with 5% defibrinated horse blood after 5 to 7 days. The concentration (CFU/ml) of the bacteria was determined by measuring the optical density (OD) at 600 nm of the bacteria suspension in KRG buffer by a spectrophotometer (BioPhotometer plus); (Eppendorf AG, Hamburg, Germany).

### *P. gingivalis* inoculation

AoSMCs from passage 5–10 were dissociated by trypsin/EDTA solution (Gibco, Carlsbad, CA). After add cell culture medium, the suspended cells were centrifuged at 14,000 rpm for 4 min and re-suspended in fresh cell culture medium. 150,000 cells were seeded per well of the 6-well plate coated with Type I collagen (Gibco, Carlsbad, CA). Cells were cultured in DMEM medium (Gibco, Carlsbad, CA) with 0.5% FBS (Sigma, St. Louis, MO), 2 mM L-glutamine (Gibco, Carlsbad, CA) and antibiotics (Gibco, Carlsbad, CA) for 24 h to get starved. Whereafter, AoSMCs were washed and re-suspended with fresh DMEM medium with 2 mM L- glutamine. The AoSMCs were challenged with different strain of *P. gingivalis* with the concentration of 10 MOI for 24 h. For DPG3 and KRX178 infection, 1 ug/ml of erythromycin were added to each well and AoSMCs, treated with 1 ug/ml erythromycin were served as control.

### Microarray gene expression analysis

The RNA from *P. gingivalis*-infected AoSMCs for 24 h was extracted from the cells using RNeasy Kit (Omega Bio-Tek, Norcross, GA). The integrity of RNA was accessed using Agilent Bioanalyze (Agilent, Santa Clara, CA) and nanodrop 2000 (Thermo, Wilmington, DE). Followed the protocol of Agilent one color microarray, the RNA samples were stained with cy3 fluorescence dye, fragmented, and load to Agilent human whole genome 8 × 60k arrays (Agilent, Santa Clara, CA). After hybridizing the slides for 17 h at 65 °C with rotation at 10× g, the array slides were scanned by Agilent Microarray Scanner (Agilent, Santa Clara, CA). The microarray data for ATCC33277 infected AoSMCs was downloaded from the Arrayexpress database (E-MTAB-1922) uploaded from our previous study^48^. The limma package^49^ offered by Bioconductor repository^50^ was used to analyze the data from scanned array pictures pre-processed by Feature Extraction software (version 6.1.1, Agilent Technologies). After normalized the data, the linear model from limma package was applied to find the differentially expressed genes between each experiment groups with the cutoff of fold change >1.5 combined with Benjamini-Hochberg false discovery rate (FDR) <0.05.

### Disease ontology, KEGG pathway, and gene ontology enrichment analysis

The lists of Entrez Gene identifiers (Entrez Gene IDs) for differentially expressed genes from each group were input into clusterProfiler^51^ R package. Through enrichDO function, the diseases associated to the interesting genes were picked out according to the moderated t-test adjusted by Benjamini-Hochberg FDR. The DO categories with adjust *p-value* less than 0.1 were identified as significantly enriched DO categories. For KEGG pathway enrichment analysis, the GeneAnswers and SPIA package from bioconductor was used to find the pathways enriched by different genes lists of interest. The ENTREZ gene ID, fold change, and adjust *p-value* for each significantly expressed genes were input GeneAnswers or SPIA package. The significant KEGG pathways were identified with false discovery adjusted global *p-value* less than 0.1 for GeneAnswers package or *p-value* less than 0.05 for SPIA package. The GO cluster analysis was performed using the functional annotation tools function of Database for Annotation Visualization, and DAVID bioinformatics tool for significantly up-regulated and down-regulated common genes regulated by three wild type *P. gingivalis* strains, respectively.

### Quantitative real-time PCR validation

After RNA was isolated from the cells, cDNA were synthesized using equal amounts of RNA by High Capacity cDNA Reverse Transcription Kits (PERkin- Elmer Applied Biosystems, Foster City, CA) according to the manufacturer’s protocol. Real-time PCR was performed using SYBR Green PCR kit (Fermentas, Sweden) with an ABI Prism 7900HT Sequence Analyzer (PERkin- Elmer Applied Biosystems, Foster City, CA). The primer sequences of 8 genes based on microarray experiment results were selected for qRT-PCR analysis and were listed in Table S12. Relative quantification of gene expression was determined by using the ΔΔCt method and normalized by the Ct value of GAPDH.

### Statistical Analysis

All experiments were preformed three times. Differentially expressed genes were identified by using Benjamini-Hochberg FDR to correct the multiple hypothesis test for the results preprocessed by limma package.

## Additional Information

**Data availability**: All microarray data were deposited into ArrayExpress database (E-MTAB- 3955). Other supporting data are available as addition files.

**How to cite this article**: Zhang, B. *et al.* Transcriptional profiling of human smooth muscle cells infected with gingipain and fimbriae mutants of *Porphyromonas gingivalis*. *Sci. Rep.* **6**, 21911; doi: 10.1038/srep21911 (2016).

## Supplementary Material

Supplementary Dataset S1

Supplementary Dataset S2

Supplementary Dataset S3

Supplementary Dataset S4

Supplementary Dataset S5

Supplementary Dataset S6

Supplementary Dataset S7

Supplementary Dataset S8

Supplementary Dataset S9

Supplementary Dataset S10

Supplementary Dataset S11

Supplementary Dataset S12

## Figures and Tables

**Table 1:** Gene ontology analysis for the common up-regulated and down-regulated genes.

Up-regulated genes			
Annotation cluster 1		Enrichment score: 7.446	
Category	Term	Count	P-value
GOTERM_BP_FAT	blood vessel development	23	3.93E-09
GOTERM_BP_FAT	vasculature development	23	6.17E-09
GOTERM_BP_FAT	blood vessel morphogenesis	21	8.17E-09
GOTERM_BP_FAT	angiogenesis	14	8.28E-06
**Down-regulated genes**			
**Annotation cluster 1**		**Enrichment score: 4.030**	
GOTERM_BP_FAT	enzyme linked receptor protein signaling pathway	24	4.52E-07
GOTERM_BP_FAT	transmembrane receptor protein tyrosine kinase signaling pathway	17	1.27E-05
GOTERM_BP_FAT	cell surface receptor linked signal transduction	45	0.1418558

## References

[b1] Strong J. P. *et al.* Prevalence and extent of atherosclerosis in adolescents and young adults: implications for prevention from the Pathobiological Determinants of Atherosclerosis in Youth Study. JAMA 281, 727–35 (1999). 10052443 10.1001/jama.281.8.727

[b2] Libby P. & Hansson G. K. Involvement of the immune system in human atherogenesis: current knowledge and unanswered questions. Lab Invest 64, 5–15 (1991). 1990208

[b3] Kume N., Cybulsky M. I. & Gimbrone M. A. Jr. Lysophosphatidylcholine, a component of atherogenic lipoproteins, induces mononuclear leukocyte adhesion molecules in cultured human and rabbit arterial endothelial cells. J Clin Invest 90, 1138–44 (1992). 1381720 10.1172/JCI115932 PMC329976

[b4] Burke-Gaffney A., Brooks A. V. & Bogle R. G. Regulation of chemokine expression in atherosclerosis. Vascul Pharmacol 38, 283–92 (2002). 12487033 10.1016/s1537-1891(02)00253-7

[b5] Lacolley P., Regnault V., Nicoletti A., Li Z. & Michel J. B. The vascular smooth muscle cell in arterial pathology: a cell that can take on multiple roles. Cardiovasc Res 95, 194–204 (2012). 22467316 10.1093/cvr/cvs135

[b6] Levi F., Lucchini F., Negri E. & La Vecchia C. Trends in mortality from cardiovascular and cerebrovascular diseases in Europe and other areas of the world. Heart 88, 119–24 (2002). 12117828 10.1136/heart.88.2.119 PMC1767229

[b7] Elkaim R. *et al.* Prevalence of periodontal pathogens in subgingival lesions, atherosclerotic plaques and healthy blood vessels: a preliminary study. J Periodontal Res 43, 224–31 (2008). 18326058 10.1111/j.1600-0765.2007.01018.x

[b8] Gibson F. C. 3rd, Yumoto H., Takahashi Y., Chou H. H. & Genco C. A. Innate immune signaling and Porphyromonas gingivalis-accelerated atherosclerosis. J Dent Res 85, 106–21 (2006). 16434728 10.1177/154405910608500202

[b9] Carramolino-Cuellar E., Tomas I. & Jimenez-Soriano Y. Relationship between the oral cavity and cardiovascular diseases and metabolic syndrome. Med Oral Patol Oral Cir Bucal 19, e289–94 (2014). 24121926 10.4317/medoral.19563 PMC4048119

[b10] Brodala N. *et al.* Porphyromonas gingivalis bacteremia induces coronary and aortic atherosclerosis in normocholesterolemic and hypercholesterolemic pigs. Arterioscler Thromb Vasc Biol 25, 1446–51 (2005). 15845905 10.1161/01.ATV.0000167525.69400.9c

[b11] Koizumi Y., Kurita-Ochiai T., Oguchi S. & Yamamoto M. Nasal immunization with Porphyromonas gingivalis outer membrane protein decreases P. gingivalis-induced atherosclerosis and inflammation in spontaneously hyperlipidemic mice. Infect Immun 76, 2958–65 (2008). 18426881 10.1128/IAI.01572-07 PMC2446728

[b12] Casarin R. C. *et al.* Levels of Aggregatibacter actinomycetemcomitans, Porphyromonas gingivalis, inflammatory cytokines and species-specific immunoglobulin G in generalized aggressive and chronic periodontitis. J Periodontal Res 45, 635–42 (2010). 20546109 10.1111/j.1600-0765.2010.01278.x

[b13] Huck O. *et al.* Evaluating periodontal risk for patients at risk of or suffering from atherosclerosis: recent biological hypotheses and therapeutic consequences. Arch Cardiovasc Dis 104, 352–8 (2011). 21693372 10.1016/j.acvd.2011.02.002

[b14] Jayaprakash K., Khalaf H. & Bengtsson T. Gingipains from Porphyromonas gingivalis play a significant role in induction and regulation of CXCL8 in THP-1 cells. BMC Microbiol 14, 193 (2014). 25037882 10.1186/1471-2180-14-193 PMC4115476

[b15] Khalaf H., Lonn J. & Bengtsson T. Cytokines and chemokines are differentially expressed in patients with periodontitis: possible role for TGF-beta1 as a marker for disease progression. Cytokine 67, 29–35 (2014). 24680479 10.1016/j.cyto.2014.02.007

[b16] Nassar H. *et al.* Role for fimbriae and lysine-specific cysteine proteinase gingipain K in expression of interleukin-8 and monocyte chemoattractant protein in Porphyromonas gingivalis-infected endothelial cells. Infect Immun 70, 268–76 (2002). 11748192 10.1128/IAI.70.1.268-276.2002 PMC127609

[b17] Palm E., Khalaf H. & Bengtsson T. Suppression of inflammatory responses of human gingival fibroblasts by gingipains from Porphyromonas gingivalis. Mol Oral Microbiol 30, 74–85 (2015). 25055828 10.1111/omi.12073

[b18] Deshpande R. G., Khan M. B. & Genco C. A. Invasion of aortic and heart endothelial cells by Porphyromonas gingivalis. Infect Immun 66, 5337–43 (1998). 9784541 10.1128/iai.66.11.5337-5343.1998 PMC108667

[b19] Lamont R. J. & Yilmaz O. In or out: the invasiveness of oral bacteria. Periodontol 2000 30, 61–9 (2002). 12236896 10.1034/j.1600-0757.2002.03006.x

[b20] Enersen M., Nakano K. & Amano A. Porphyromonas gingivalis fimbriae. J Oral Microbiol 5, 20265 (2013). 10.3402/jom.v5i0.20265 PMC3647041 23667717

[b21] Shoji M. *et al.* The major structural components of two cell surface filaments of Porphyromonas gingivalis are matured through lipoprotein precursors. Mol Microbiol 52, 1513–25 (2004). 15165251 10.1111/j.1365-2958.2004.04105.x

[b22] Liu Z. J. *et al.* Notch activation induces endothelial cell senescence and pro-inflammatory response: implication of Notch signaling in atherosclerosis. Atherosclerosis 225, 296–303 (2012). 23078884 10.1016/j.atherosclerosis.2012.04.010 PMC3502717

[b23] Aquila G. *et al.* The role of Notch pathway in cardiovascular diseases. Glob Cardiol Sci Pract 2013, 364–71 (2013). 24749110 10.5339/gscp.2013.44 PMC3991209

[b24] Rosenfeld M. E. & Campbell L. A. Pathogens and atherosclerosis: update on the potential contribution of multiple infectious organisms to the pathogenesis of atherosclerosis. Thromb Haemost 106, 858–67 (2011). 22012133 10.1160/TH11-06-0392

[b25] Buhlin K. *et al.* Risk factors for atherosclerosis in cases with severe periodontitis. J Clin Periodontol 36, 541–9 (2009). 19538326 10.1111/j.1600-051X.2009.01430.x

[b26] Yakob M. *et al.* Prevotella nigrescens and Porphyromonas gingivalis are associated with signs of carotid atherosclerosis in subjects with and without periodontitis. J Periodontal Res 46, 749–55 (2011). 21793826 10.1111/j.1600-0765.2011.01398.x

[b27] Potempa J., Pike R. & Travis J. Titration and mapping of the active site of cysteine proteinases from Porphyromonas gingivalis (gingipains) using peptidyl chloromethanes. Biol Chem 378, 223–30 (1997). 9165075 10.1515/bchm.1997.378.3-4.223

[b28] Imamura T. The role of gingipains in the pathogenesis of periodontal disease. J Periodontol 74, 111–8 (2003). 12593605 10.1902/jop.2003.74.1.111

[b29] Takahashi Y., Davey M., Yumoto H., Gibson F. C. 3rd & Genco C. A. Fimbria-dependent activation of pro-inflammatory molecules in Porphyromonas gingivalis infected human aortic endothelial cells. Cell Microbiol 8, 738–57 (2006). 16611224 10.1111/j.1462-5822.2005.00661.x

[b30] Yoshimura F., Murakami Y., Nishikawa K., Hasegawa Y. & Kawaminami S. Surface components of Porphyromonas gingivalis. J Periodontal Res 44, 1–12 (2009). 18973529 10.1111/j.1600-0765.2008.01135.x

[b31] Marchesan J. T. *et al.* Porphyromonas gingivalis oral infection exacerbates the development and severity of collagen-induced arthritis. Arthritis Res Ther 15, R186 (2013). 24456966 10.1186/ar4376 PMC3979094

[b32] Mikuls T. R. *et al.* Periodontitis and Porphyromonas gingivalis in patients with rheumatoid arthritis. Arthritis Rheumatol 66, 1090–100 (2014). 24782175 10.1002/art.38348 PMC4115329

[b33] Gerthoffer W. T. Mechanisms of vascular smooth muscle cell migration. Circ Res 100, 607–21 (2007). 17363707 10.1161/01.RES.0000258492.96097.47

[b34] Disanza A. *et al.* Actin polymerization machinery: the finish line of signaling networks, the starting point of cellular movement. Cell Mol Life Sci 62, 955–70 (2005). 15868099 10.1007/s00018-004-4472-6 PMC11924564

[b35] Simcock D. E. *et al.* Induction of angiogenesis by airway smooth muscle from patients with asthma. Am J Respir Crit Care Med 178, 460–8 (2008). 18556625 10.1164/rccm.200707-1046OC

[b36] Louis S. F. & Zahradka P. Vascular smooth muscle cell motility: From migration to invasion. Exp Clin Cardiol 15, e75–85 (2010). 21264073 PMC3016065

[b37] Johnson J. L. Emerging regulators of vascular smooth muscle cell function in the development and progression of atherosclerosis. Cardiovasc Res 103, 452–60 (2014). 25053639 10.1093/cvr/cvu171

[b38] Lin G. *et al.* Effects of micro-amounts of Porphyromonas gingivalis lipopolysaccharide on rabbit inflammatory immune response and development of atherosclerosis. J Periodontal Res 50, 356–62 (2015). 25065326 10.1111/jre.12214

[b39] Muslin A. J. MAPK signalling in cardiovascular health and disease: molecular mechanisms and therapeutic targets. Clin Sci (Lond) 115, 203–18 (2008). 18752467 10.1042/CS20070430 PMC2707780

[b40] Khalaf H., Demirel I. & Bengtsson T. Suppression of inflammatory gene expression in T cells by Porphyromonas gingivalis is mediated by targeting MAPK signaling. Cell Mol Immunol 10, 413–22 (2013). 23892429 10.1038/cmi.2013.23 PMC4003196

[b41] Meyer M. S., Joshipura K., Giovannucci E. & Michaud D. S. A review of the relationship between tooth loss, periodontal disease, and cancer. Cancer Causes Control 19, 895–907 (2008). 18478344 10.1007/s10552-008-9163-4 PMC2723958

[b42] Mager D. L. *et al.* The salivary microbiota as a diagnostic indicator of oral cancer: a descriptive, non-randomized study of cancer-free and oral squamous cell carcinoma subjects. J Transl Med 3, 27 (2005). 15987522 10.1186/1479-5876-3-27 PMC1226180

[b43] Katz J., Onate M. D., Pauley K. M., Bhattacharyya I. & Cha S. Presence of Porphyromonas gingivalis in gingival squamous cell carcinoma. Int J Oral Sci 3, 209–15 (2011). 22010579 10.4248/IJOS11075 PMC3469978

[b44] Michaud D. S. *et al.* Plasma antibodies to oral bacteria and risk of pancreatic cancer in a large European prospective cohort study. Gut 62, 1764–70 (2013). 22990306 10.1136/gutjnl-2012-303006 PMC3815505

[b45] Ahn J., Segers S. & Hayes R. B. Periodontal disease, Porphyromonas gingivalis serum antibody levels and orodigestive cancer mortality. Carcinogenesis 33, 1055–8 (2012). 22367402 10.1093/carcin/bgs112 PMC3334514

[b46] Mitka M. Evidence lacking for benefit from oral cancer screening. JAMA 309, 1884 (2013). 23652503 10.1001/jama.2013.4913

[b47] Zhang B., Khalaf H., Sirsjo A. & Bengtsson T. Gingipains from the periodontal pathogen Porphyromonas gingivalis play a significant role in regulation of Angiopoietin 1 and Angiopoietin 2 in human aortic smooth muscle cells. Infect Immun 83, 4256–65 (2015). 26283334 10.1128/IAI.00498-15 PMC4598411

[b48] Zhang B. *et al.* The periodontal pathogen Porphyromonas gingivalis changes the gene expression in vascular smooth muscle cells involving the TGFbeta/Notch signalling pathway and increased cell proliferation. BMC Genomics 14, 770 (2013). 24209892 10.1186/1471-2164-14-770 PMC3827841

[b49] Bolstad B. M., Irizarry R. A., Astrand M. & Speed T. P. A comparison of normalization methods for high density oligonucleotide array data based on variance and bias. Bioinformatics 19, 185–93 (2003). 12538238 10.1093/bioinformatics/19.2.185

[b50] Gentleman R. C. *et al.* Bioconductor: open software development for computational biology and bioinformatics. Genome Biol 5, R80 (2004). 15461798 10.1186/gb-2004-5-10-r80 PMC545600

[b51] Yu G., Wang L. G., Han Y. & He Q. Y. clusterProfiler: an R package for comparing biological themes among gene clusters. OMICS 16, 284–7 (2012). 22455463 10.1089/omi.2011.0118 PMC3339379

