# Supplementary material for: Transcriptional profiling of human smooth muscle cells infected with gingipain and fimbriae mutants of Porphyromonas gingivalis
Source: Sci Rep. 2016 Feb 24;6:21911. doi: 10.1038/srep21911 (PMC4764818; doi:10.1038/srep21911)
Supplement: Supplementary Dataset S12 [file srep21911-s12.doc]

**Transcriptional profiling of human smooth muscle cells infected with gingipain and fimbriae mutants of *Porphyromonas gingivalis***

**Boxi Zhang *, Allan Sirsjö, Hazem Khalaf, Torbjörn Bengtsson**

Table S12. Primer sequences for real-time PCR

| **Gene** | **Forward primer** | **Reverse primer** |
| --- | --- | --- |
| **CCL11** | GCTACAGGAGAATCACCAGTGG | GGAATCCTGCACCCACTTCTTC |
| **IL-7** | GACAGCATGAAAGAAATTGGTAGC | CAACTTGCGAGCAGCACGGAAT |
| **NOD1** | CAACGGCATCTCCACAGAAGGA | CCAAACTCTCTGCCACTTCATCG |
| **IL-1α** | TGTATGTGACTGCCCAAGATGAAG | AGAGGAGGTTGGTCTCACTACC |
| **Angpt2** | ATTCAGCGACGTGAGGATGGCA | GCACATAGCGTTGCTGATTAGTC |
| **NOTCH1** | GGTGAACTGCTCTGAGGAGATC | GGATTGCAGTCGTCCACGTTGA |
| **CX3CL1** | ACAGCACCACGGTGTGACGAAA | AACAGCCTGTGCTGTCTCGTCT |
| **CXCL8** | GAGAGTGATTGAGAGTGGACCAC | CACAACCCTCTGCACCCAGTTT |
